# In vitro suppression of inflammatory cytokine response by methionine sulfoximine

**DOI:** 10.1186/s12950-018-0193-8

**Published:** 2018-09-10

**Authors:** Tyler J. Peters, Amruta A. Jambekar, William S. A. Brusilow

**Affiliations:** 1 Department of Biochemistry and Molecular Biology, Wayne State University School of Medicine, Scott Hall, 540 E. Canfield Ave, Detroit, MI 48201 USA; 2 Burtch Pharmaceuticals LLC, Detroit, MI USA

## Abstract

**Background:**

The glutamine synthetase inhibitor methionine sulfoximine (MSO), shown previously to prevent death caused by an inflammatory liver response in mice, was tested on in vitro production of cytokines by mouse peritoneal macrophages triggered with lipopolysaccharide (LPS).

**Results:**

MSO significantly reduced the production of Interleukin 6 (IL-6) and Tumor Necrosis Factor Alpha (TNFα) at 4 and 6 h after LPS-treatment. This reduction did not result from decreased transcription of IL-6 and TNFα genes, and therefore appeared to result from post-transcriptional inhibition of synthesis of these cytokines. MSO treatment did not inhibit total protein synthesis and did not reduce the production of a third LPS-triggered cytokine CXCL1, so the effect was not a toxic or global downregulation of the LPS response. The anti-inflammatory effects of a glutamine synthetase inhibitor were seen even though the medium contained abundant (2 mM) glutamine, suggesting that the target for this activity was not glutamine synthetase. In agreement with this hypothesis, the L,*R* isomer of MSO, which does not inhibit glutamine synthetase and was previously thought to be inert, both significantly reduced IL-6 secretion in isolated macrophages and increased survival in a mouse model for inflammatory liver failure.

**Conclusions:**

Our findings provide evidence for a novel target of MSO. Future attempts to identify the additional target would therefore also provide a target for therapies to treat diseases involving damaging cytokine responses.

## Background

Methionine sulfoximine (MSO) is an amino acid inhibitor of glutamine synthetase (GS) [1–3]. In addition to its historical use in dissecting the mechanism of GS, this molecule has also been shown to have therapeutic effects in animal models of hepatic encephalopathy, amyotrophic lateral sclerosis (ALS), and inflammatory liver failure [4]. For hepatic encephalopathy, MSO treatment reduces glutamine in astrocytes, preventing the osmotic swelling that results from ammonia-driven synthesis of glutamine from glutamate [5]. For ALS, it appears that MSO reduces excitotoxicity by reducing the levels of both glutamine and glutamate in the brain [6, 7]. Additionally, MSO appears to have anti-inflammatory effects, since it increases survival in a mouse model for inflammatory liver failure resulting from exposure to lipopolysaccharide (LPS) [8]. MSO reduces the LPS-induced cytokine response for several inflammatory cytokines, most notably tumor necrosis factor alpha (TNFα) and interleukin 6 (IL-6), two cytokines strongly associated with an array of inflammatory diseases [9, 10]. It was previously unclear to what extent these effects are mediated by glutamine synthetase inhibition, or if there is another target for this molecule.

In order to determine the anti-inflammatory target(s) for MSO, we measured its effects on the cytokine response of isolated macrophages to LPS treatment, its effects on transcription of cytokine genes, the role of medium glutamine in MSO-inhibition of the cytokine response, and the effects of both the L,*R* and L,*S* stereoisomers of MSO on this anti-inflammatory action in vivo and in vitro.

## Methods

### Ethics

All animal experiments complied with Institutional Animal Care and Use Committee (IACUC) guidelines for animal welfare at Wayne State University. Male CD1 mice were purchased from Charles River (USA). All animals used for experiments were 6–10 weeks old.

### Rat Kupffer cell culture

Cryopreserved Rat Kupffer cells (ThermoFisher Scientific, USA) were thawed in a 37 °C water bath before being gently pipetted into 8 mL of ice cold Advanced DMEM Medium (ThermoFisher Scientific) containing 5% FBS (Gibco, USA) and 4% Thawing and Plating Cocktail A (Catalog No. CM3000, ThermoFisher Scientific). Cells were centrifuged at 500 x g for 10 min at 4 °C. The supernatant was discarded, and the cells were resuspended in 2 mL of medium and counted using a hemocytometer. Cells were seeded into 12-well plates at 4 × 10^5^ cells per well and placed into a humidified incubator with 95% air, 5% CO^2^ at 37 °C. 4 h later the media was aspirated and cells were washed 3 times with warm PBS before 1 mL of RPMI 1640 with GlutaMAX™ supplement and HEPES (ThermoFisher Scientific), containing 10% FBS and a 1% mixture of penicillin-streptomycin (ThermoFisher Scientific) was added to each well. Cells were incubated overnight (20–24 h) before any experiments were performed.

### Mouse peritoneal macrophage culture

Peritoneal macrophages were isolated as previously described by Zhang et al. (2008) with minor modifications [11]. Two mice were used for each experiment. After euthanasia, 10 mL of cold phosphate buffered saline (4 °C) was injected into the peritoneal cavities of each mouse. The abdomens were gently massaged before a small incision was made within the abdominal wall and the exudate was aspirated using a disposable transfer pipette and transferred into a 15 mL conical tube. Cell suspensions were centrifuged at 500 x g for 10 min at 4^0^ C. The supernatant was discarded, and the pellet was gently resuspended in 2 mL of Advanced DMEM Medium containing 5% FBS and 4% Thawing and Plating Cocktail A on ice. Approximately 2 × 10^5^ cells were added to each well of a 12-well tissue culture plate which was placed in a humidified incubator with 5% CO^2^ for 2 h at 37 °C. The media was aspirated, and cells were washed three times with warmed PBS before 1 mL of RPMI 1640 with GlutaMAX™ supplement and HEPES, containing 10% FBS and a 1% mixture of penicillin-streptomycin was added to each well. Cells were incubated overnight (20–24 h) before any experiments were performed.

### Macrophage activation

All media and reagents were sterile filtered through a 0.22 μm syringe filter prior to use. L-Methionine R,S-sulfoximine (Sigma, USA), and Lipopolysaccharides from *Escherichia coli* O111:B4 (Catalog No. L6230, Sigma), were dissolved in sterile PBS. Purified L,*S* and L,*R* MSO were purchased from Toronto Research Chemicals (Canada). Cells were divided into four treatments each with at least two biological replicates. Treatments consisted of LPS only, MSO + LPS, MSO only, and untreated. All cells in culture were washed 2–3 times with warm, serum-free RPMI 1640 with GlutaMAX™ supplement and HEPES before 0.5 mL of media, with or without MSO, was added to each well. After incubation for one hour, LPS (1 μg/mL) was added to the appropriate wells, and media samples were collected at different times for the measurements described in the different experiments.

### Intracellular IL-6 measurement

For intracellular protein quantification, the medium was removed, and cells were lysed directly within wells of the cell culture plate in 20 mM Tris buffer containing 100 mM NaCl, 1 mM EDTA, and 0.2% Triton X-100 (pH 7.4). After 20 min of incubation on ice, cells were scraped vigorously and observed under the microscope to ensure complete lysis. IL-6 within cell lysates was measured using the Mouse IL-6 Ready-SET-Go® ELISA kit (eBioscience, USA).

### IL-6 immunofluorescence

Cells were cultured on 8-well glass chamber slides (Millicell® EZ SLIDE 8-well glass, sterile, Catalog No. PEZGS0816, Millipore, USA) in the same manner as described for the experiments performed in tissue culture plates. Cells were pre-treated with 9 mM MSO for one hour prior to the addition of 1 μg/kg LPS. Cells treated with LPS alone served as positive controls, whereas cells treated with MSO alone or left untreated served as negative controls. After 6 h of LPS stimulation, the medium was aspirated, and cells were washed three times with PBS before being fixed to the chamber slides with a 4% paraformaldehyde solution in PBS for 10 min. Cells were made permeable by the addition of a 0.1% Triton X-100 solution in PBS for 20 min, followed by an overnight incubation with a monoclonal antibody specific for mouse IL-6 (Cell signaling technology, USA, Catalog No. D5W4V) at 4 degrees. Cells were washed before incubation with an Alexa Fluor-conjugated secondary antibody (Abcam, USA, Catalog No. ab15007) in the presence of 10% goat serum (R&D systems, USA). A mounting medium containing the nuclear stain DAPI (Electron Microscopy Sciences, USA, Catalog No. 17985–50) was applied to a cover slip, and fluorescence was visualized under a confocal microscope.

### Glutamine depletion experiments

Cells were isolated and plated in 12-well plates in the conditions listed above. After overnight incubation, cells were washed 3 times with RPMI either containing glutamine or RPMI without glutamine. 0.5 mL of fresh, serum-free medium, with or without glutamine and MSO was added to each well one hour prior to the addition of LPS. Experiments were limited to 6 h in consideration of the possibility that cell death could occur due to prolonged glutamine depletion.

### Enzyme linked immunosorbent assay (ELISA)

Mouse and Rat TNF-alpha Ready-SET-Go® ELISA kits, and Mouse IL-6 Ready-SET-Go® ELISA kits (eBioscience, USA) were used to assess the levels of TNFα and IL-6 in the macrophage activation experiments. Mouse CXCL1/KC DuoSet ELISA (R&D systems, USA) was used to measure mouse CXCL1 in all macrophage activation experiments. The procedures were carried out per the manufactures instructions, and all data were analyzed using the Epoch microplate spectrophotometer and Gen5 data analysis software (Biotek, USA).

### Lactate dehydrogenase (LDH) assay

LDH release from peritoneal macrophages was assessed with the Cytotoxicity Detection Kit (LDH) (Sigma Aldrich, USA) per the instructions provided by the manufacturer. For this assay, LDH activity is proportional to colorimetric reduction of tetrazolium salt measured at 490 nm. To serve as a positive control, a subset of cells was exposed to a Triton X-100 solution for 45 min, and the average OD 490 nm measurement from these cells was considered as the maximum LDH activity (100%) and served to normalize LDH values obtained from cells treated with MSO. An additional subset of cells treated with dH_2_O, served as a negative control.

### RNA isolation and reverse transcription PCR (RT-PCR)

Approximately 2 × 10^6^ cells per well were plated in 6-well plates after peritoneal exudate cells were isolated in the media conditions (with or without MSO) and incubation times listed above. At 1, 3, or 5 h after LPS addition cells were lysed and RNA was isolated using the contents provided within the RNeasy mini kit (Qiagen, Germany). The RNA concentration in each sample was measured with a Qubit® fluorometer, using the RNA high sensitivity kit (Thermofisher Scientific Catalog No. Q32852). Reverse transcription reaction was performed using the iScriptTM cDNA Synthesis Kit (Bio-Rad, USA). 250 ng of total RNA were used in each 20 uL reaction, with equal amounts of total RNA used for each sample.

### Quantitative real-time PCR (qRT-PCR)

qRT-PCR was performed using a ready-to-use PowerUp™ SYBR® Green Master Mix (Thermofisher Scientific). SYBR green fluorescence was detected by the 7500 Real Time PCR System (Applied Biosystems), using 25 ng of cDNA per reaction. Gene expression was normalized to the expression of the reference gene, beta-actin (β-actin). Oligonucleotide primers for TNFα and IL-6 were previously designed and used by Simpson, Tomkins and Cooper (1997) [12]. Primer sequences were purchased as lyophilized powder (Invitrogen, USA) and are as follows: IL-6 (forward: 5’-GAC AAA GCC AGA GTC CTT CAG AGA G-3′ and reverse: 5’-CTA GGT TTG CCG AGT AGA TCT C-3′), TNFα (forward: 5′-ATG AGC ACA GAA AGC ATG ATC-3′ and reverse: 5’-TAC AGG CTT GTC ACT CGA ATT-3′), β-actin (forward: 5′-GTG GGC CGC TCT AGG CAC CA-3′ and reverse: 5’-CGG TTG GCC TTA GGG TTC AGG GGG G-3′). The quality of each PCR product was confirmed by melting curve analysis. Relative quantification of fold induction of TNFα and IL-6 mRNA was assessed using the ΔΔCt method described by Livak and Schmittgen (2001) [13].

### Radioactive protein synthesis

Peritoneal macrophages were incubated under the conditions listed above. At the start of the experiment 50 μCi of S^35^ labeled cocktail of methionine and cysteine was added to the medium. Samples were collected at 0, 1, 2, 4, 6, and 8 h after S^35^, LPS, and/ or MSO addition. For secreted protein measurements, 50 μL of medium was used. For cellular protein synthesis measurements, the cells were washed with PBS, scraped and harvested. 5 mL of 10% TCA was added to each sample, and tubes were incubated on ice for 10 min. TCA precipitated protein was filtered through glass fiber filter paper. The filter papers were rinsed twice with 5 mL of 10% TCA. Filter papers were dried and TCA-precipitated counts of S^35^ labeled proteins were measured in a Beckman LS6500 liquid scintillation counter.

### Acute liver failure mouse model

We used an accepted method to induce acute liver failure into mice by co-injecting LPS (20 μg/kg) and D-galactosamine (800 mg/kg, Sigma, USA) intraperitoneally (IP). 3 h prior to injecting LPS/D-Gal, mice were injected IP with either saline (negative control), L,*S* MSO (25 mg/kg), or L*,R* MSO (25 mg/kg). Food was removed from the cages prior to any injections, but mice were allowed access to water throughout the duration of the experiment. Mice that failed to respond to prodding and right themselves were euthanized. Survival was monitored for 24 h.

### Statistical analysis

Values are expressed as mean ± standard error (*s ±* SEM). All experiments were repeated at least three times. T-tests were performed for analyses of statistical significance in cell culture experiments. For the mouse experiments, differences in survival between groups were assessed using Fisher’s exact test. All statistical analyses were performed using IBM SPSS Statistics version 24 for Windows. Differences between treatments were considered significant when *p* < 0.05.

## Results

We have previously shown that treatment of mice with MSO reduces the LPS-induced production of cytokines TNFα, IL-6, and IFγ in vivo [8]. In this report, we describe the effects of MSO on IL-6 and TNFα production by isolated macrophages treated with LPS.

### Effect of MSO on IL-6 production

**Fig. 1:**
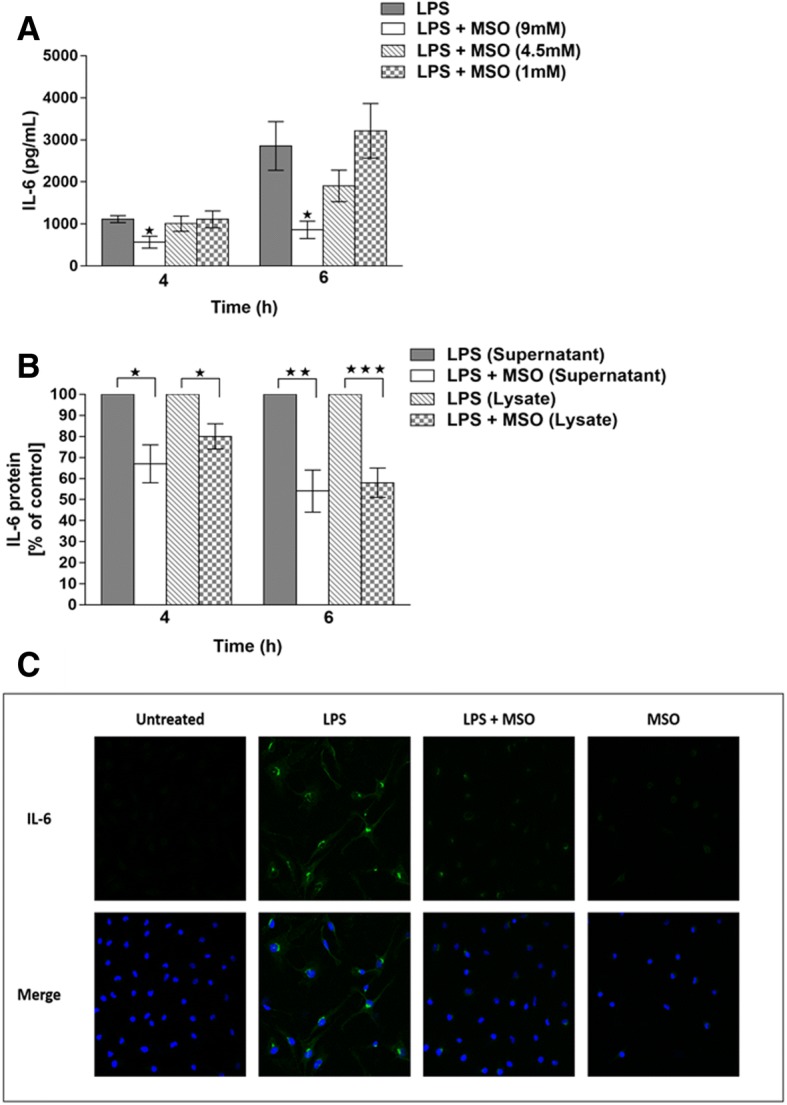
**a**, dose-dependent effects of MSO on LPS-triggered IL-6 production (s ± SEM). Peritoneal macrophages were treated with three concentrations of MSO one hour prior to the addition of 1 μg/mL LPS, and IL-6 production in the medium was quantitated by ELISA 4 h and 6 h after LPS treatment (*n* = 6, *p < 0.05). **b**, effects of MSO on intracellular and extracellular IL-6 production. Cells were treated with 9 mM MSO, and in addition to measuring IL-6 in the medium after LPS treatment, cells were washed, lysed, and the amount of IL-6 in the lysate was quantitated as a measure of intracellular IL-6. Untreated cells, or cells treated with MSO alone (minus-LPS) did not produce detectable levels of IL-6. ELISA values obtained from LPS stimulated control (minus-MSO) samples were normalized as 100%, and the average amounts of IL-6 produced in the treated supernatant and lysate samples are indicated as percentages of the controls. These data represent averages from 3 additional macrophage preps different from those represented in A, with three biological replicates for each treatment. *p < 0.05, **p < 0.01, ***p < 0.001. C, immunofluorescence detection of intracellular IL-6 protein, 6 h after LPS treatment. The top row shows, from left to right, levels of intracellular IL-6 in untreated cells, cells treated with LPS, treated with LPS and MSO, or treated with just MSO. The same cells were also stained with the nuclear stain DAPI, and the two fluorescent images were merged in the bottom row, showing that the nuclei of treated and untreated cells appear the same

We first determined the minimal effective concentration for inhibiting the LPS-triggered IL-6 response. by treating cells cultured in normal medium (2 mM glutamine) with either 9 mM, 4.5 mM, or 1 mM MSO for one hour before the addition of LPS (1 μg/mL) to the culture medium. Figure 1a shows that in two independent experiments, 9 mM MSO appeared to be the minimum effective concentration required to significantly reduce IL-6 in the culture medium of LPS-stimulated mouse peritoneal macrophages at 4 and 6 h after LPS exposure. These data suggest that a dose-dependent relationship might exist, with 9 mM MSO being the most effective in reducing IL-6 secretion, 1 mM being ineffective, and 4.5 mM falling in between. IL-6 levels in the culture medium of untreated cells or cells treated with MSO alone were undetectable (not shown).

### Effect of MSO on cellular and secreted IL-6

The observed effect of MSO on the production of secreted IL-6 resulted from either decreased synthesis or decreased secretion. We therefore compared IL-6 levels in the culture medium to IL-6 levels measured in cell lysates. IL-6 levels measured in the medium were much higher than in the cell lysates for all samples. 4 h after LPS treatment, the amount of IL-6 in the supernatant was approximately 3 times greater in the supernatant than in the lysate (3.8X for LPS-only controls and 2.9X for MSO-treated cells). At 6 h, the IL-6 measured in the supernatant was over 10 times greater than inside the cells (16.6X for LPS-only controls and 11.2X for MSO-treated cells).

Figure 1b shows that, when compared to controls, the % decreases of IL-6 resulting from MSO-treatment were similar in both the medium (secreted) and the cell lysate (intracellular). MSO treatment reduced secreted and intracellular IL-6 concentrations at both 4 and 6 h after LPS addition. At 4 h, a mean 33% reduction of IL-6 was observed in the medium of MSO treated cells, compared to a mean 20% reduction of IL-6 in the lysates. At 6 h, a mean 45% reduction of IL-6 was observed in the medium of MSO treated cells compared to a mean 42% reduction of IL-6 in the lysates. MSO-treatment therefore did not result in cells “filling up” with cytokine.

To further demonstrate that the decrease in medium IL-6 could be attributed to decreased levels of IL-6 within the cells, we used immunofluorescence to visualize IL-6 in MSO-treated and untreated cells 6 h after LPS treatment (Fig. 1c). In agreement with the results shown in Fig. 1b, Fig. 1c demonstrates a clear reduction of cytoplasmic IL-6 with MSO treatment when compared to cells treated with LPS only.

### Effect of MSO on production of TNFα

**Fig. 2:**
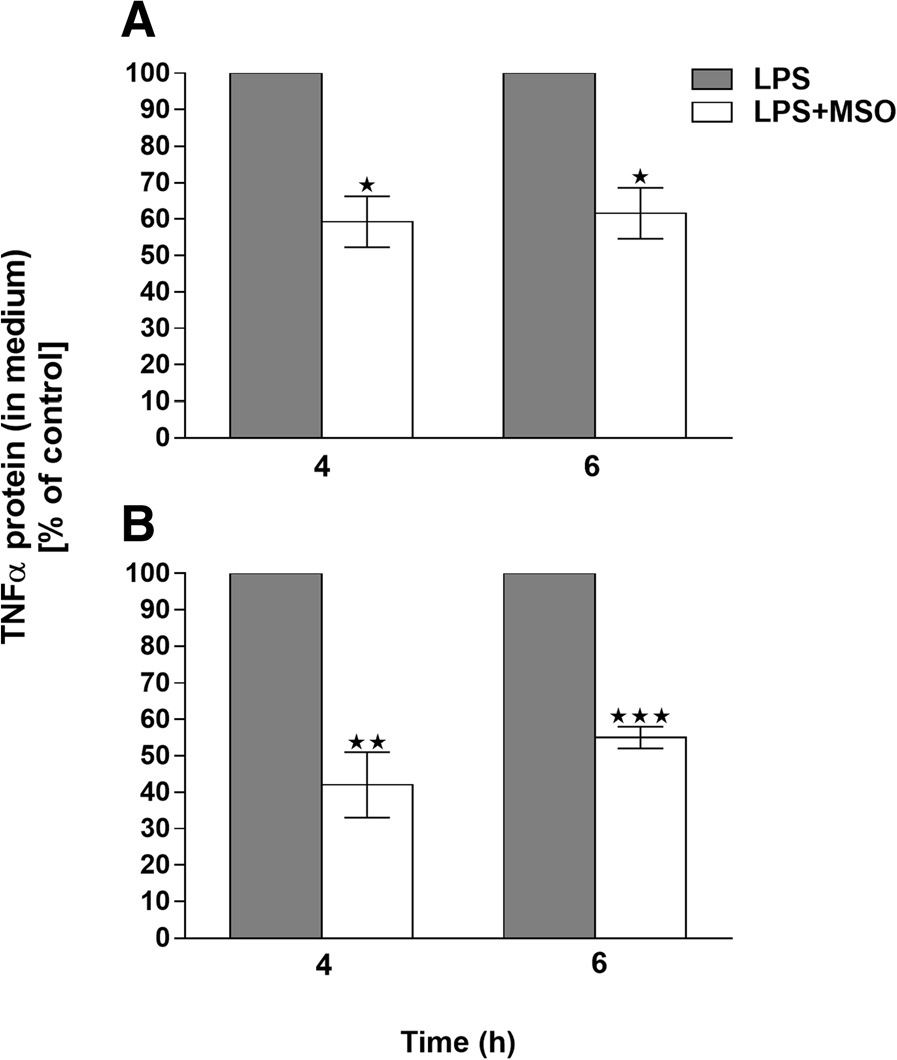
TNFα secretion by (**a**) primary mouse peritoneal macrophages and (**b**) primary rat Kupffer cells after incubation in the presence or absence of MSO and LPS. Untreated cells, or cells treated with MSO alone (minus-LPS) did not produce detectable levels of TNFα. The level of TNFα induction by LPS stimulated controls, was normalized to 100%. A, mean TNFα protein concentration in LPS-stimulated controls was 114 pg/mL at 4 h and 183 pg/mL at 6 h. **b**, mean TNFα protein concentration of LPS stimulated controls was 71 pg/mL at 4 h and 117 pg/mL at 6 h. These data represent 3 peritoneal macrophage preparations and 5 Kupffer cell preparations. Each preparation contained at least 2 biological replicates for each treatment. **p < 0.01, ***p < 0.001

Peritoneal macrophages also produce TNFα after LPS stimulation. Figure 2a shows LPS-stimulated production of TNFα in cells treated with MSO after 4, and 6 h. We found no significant effects after 2 h (not shown). Compared to cells treated with LPS alone, cells pre-treated with MSO showed significant reductions in TNFα secreted into the culture medium at 4 h (reduced by 40 ± 7%) and 6 h (reduced by 38% ± 8%)**.**

As an added test of the ability of MSO to inhibit TNF-a production, we carried out these same assays in commercially available rat Kupffer cells (see Methods). Kupffer cells are the resident liver macrophages responsible for inflammatory liver failure [14]. They respond to LPS by producing cytokines including IL-6 and TNFα. Rat Kupffer cells are commercially available, but when we tested the effects of MSO on cytokine production by those cells, we found batch-to-batch variability in the LPS-triggered responses. Most of the preparations that we purchased (five out of eight) responded to LPS treatment and produced TNFα, as they should. In those experiments, we could observe a significant decrease in TNFα secretion when these cells were pre-treated with MSO. Fig. 2b shows inhibition by MSO of LPS-triggered TNFα production in primary rat Kupffer cell cultures (*s ±* SEM), with 58% ± 9% and 45% ± 3% mean decreases in TNFα secretion after 4 and 6 h, respectively.

However, several batches of these commercially-available cells (three out of eight) did not respond to LPS, indicating that these cell preparations were of poor quality. The substantial batch-to-batch variability led us to concentrate instead on the peritoneal macrophage preparations for essentially all of our studies besides those shown in Fig. 2b, which shows the results obtained only from those batches of cells that demonstrated significant production of TNFα when treated with LPS alone.

### Effect of MSO on transcription of TNFα and IL-6

**Fig. 3:**
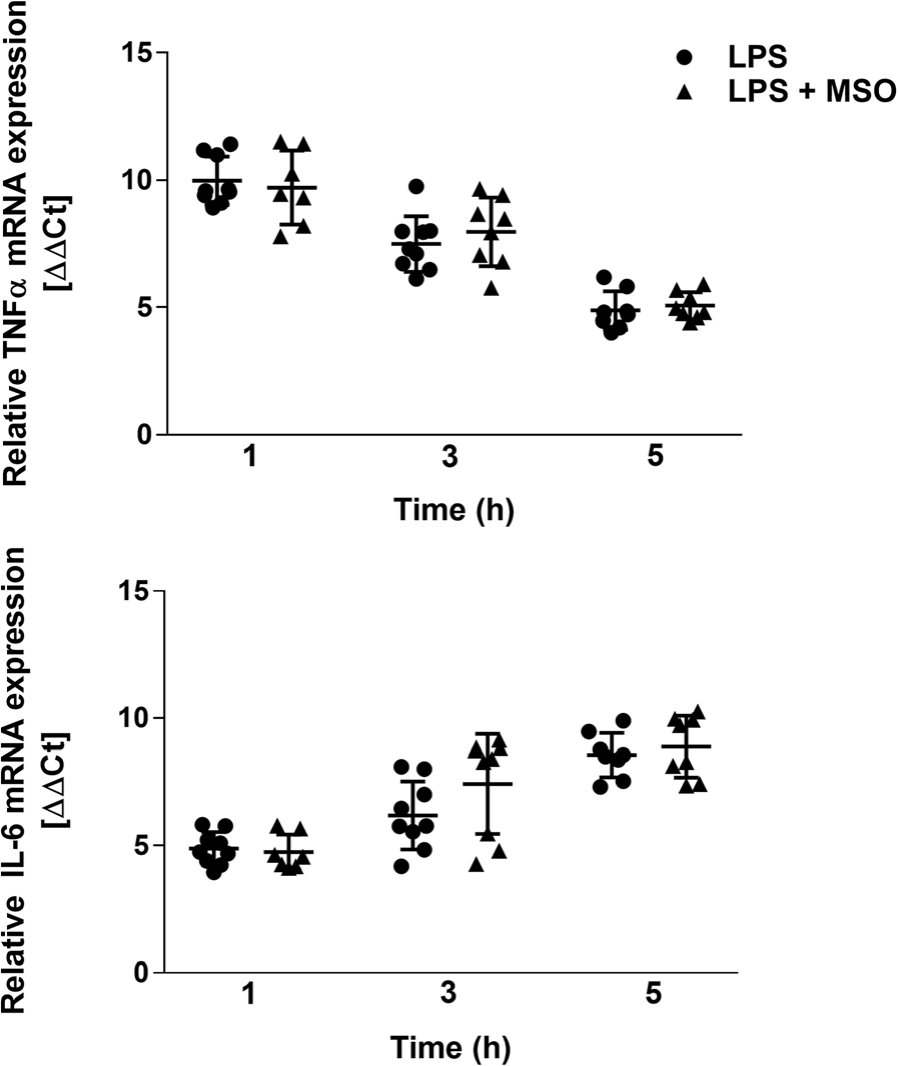
Effects of MSO on transcription of the cytokine genes. Mouse peritoneal macrophages were treated with MSO, then LPS as indicated for the experiments in Fig. 1 and Fig. 2. At 1, 3, or 5 h after LPS treatment, cells were, harvested, lysed, and mRNA was isolated as described in Methods. qRT-PCR was conducted using primers specific for either TNFα or IL-6. Relative mRNA levels are plotted as ΔΔCt, the log_2_ transformed fold increase in mRNA compared to untreated cells [13]. Data from each timepoint were obtained from at least 3 experiments, with all technical replicates shown for clarity

The expression of the genes for TNFα and IL-6 has been shown to peak between 1 and 3 h after LPS stimulation in mouse peritoneal macrophages [12]. To test if MSO affected transcription of TNFα or IL-6, cells were treated with LPS for up to 5 h, then lysed and the mRNA was isolated, reverse-transcribed into cDNA, and subjected to quantitative PCR, with relative β-actin level serving as an endogenous control. Both IL-6 and TNFα mRNA levels were unaffected by MSO (Fig. 3), suggesting that the inhibitory action of MSO on the production of these proteins does not occur at the level of gene expression.

### Effect of MSO on protein synthesis and production of the cytokine CXCL1

**Fig. 4:**
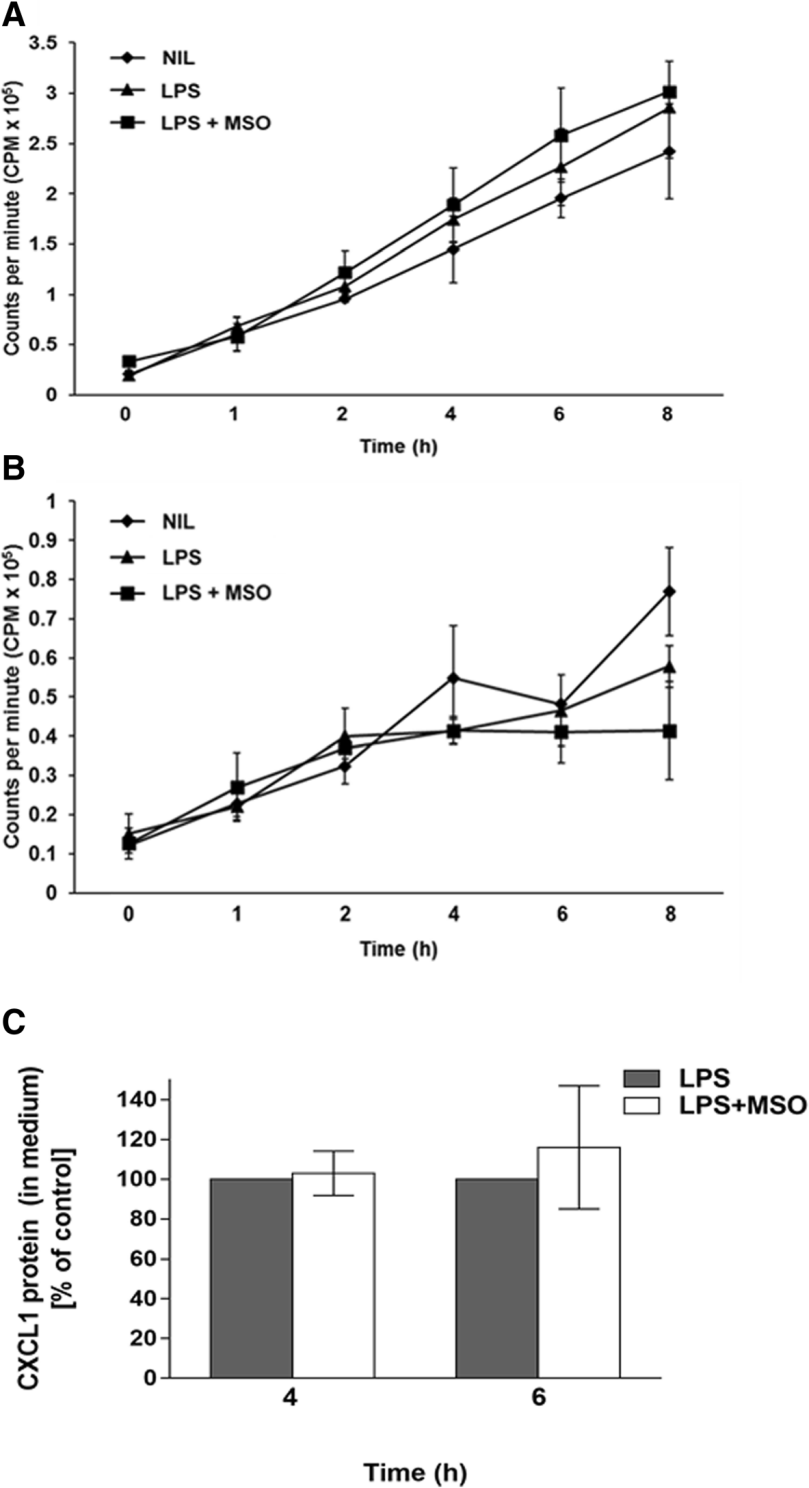
Effects of MSO on protein synthesis and on LPS-triggered production of the chemokine CXCL1. (**a**, **b**) mouse peritoneal macrophages were treated as described in Fig. 1 in medium containing 50 μCi of a mixture of S^35^-labeled methionine and cysteine. At the indicated times after LPS treatment, (**a**) cells were harvested, TCA-precipitated, filtered and counted, and at the same times (**b**) supernatant was collected, TCA precipitated, filtered, and counted to determine how overall levels of **a***,* cellular, and **b**, secreted protein synthesis was affected by MSO treatment shown to reduce production of IL-6 and TNFα. Data shown (s ± SEM) represent 3 macrophage preparations. **c**, effect of MSO on LPS-triggered production of the cytokine CXCL1 (*n* = 3). Experiments were carried out as described for Fig. 1 and Fig. 2. ELISA was used to quantitate LPS-triggered production of CXCL1 in the presence or absence of MSO 4 h and 6 h after LPS treatment. CXCL1 was not detectable in the medium of untreated cells, or for cells treated with MSO alone (minus-LPS). The mean CXCL1 protein concentration measured in LPS stimulated controls was 450 pg/mL at 4 h and 750 pg/mL at 6 h. Control values were normalized to 100%, and average values from treated samples were plotted in comparison to those controls

Although MSO concentrations exceeding 9 mM have been used in other cell culture models without affecting cell viability [15–17], we tested whether 9 mM MSO was having a global effect on protein synthesis in our system. Cells were isolated and treated with LPS in medium containing S^35^-labelled methionine and cysteine. Samples were taken from either the cells alone or from the culture supernatant and TCA-precipitated to measure the incorporation of radioactivity into proteins in the presence or absence of 9 mM MSO. Fig. 4a shows total cellular protein synthesis in peritoneal macrophages, and Fig. 4b shows proteins synthesized and secreted into the culture medium, as measured by the incorporation of S^35^ labeled methionine and cysteine. The presence of MSO did not affect the incorporation of radioactivity into either cellular proteins or secreted proteins. Cells treated with MSO alone did not demonstrate reduced incorporation of radioactivity (not shown for graph clarity).

Furthermore, we tested the effect of MSO treatment on the production of another cytokine produced when cells are stimulated with LPS. CXCL1 is a small cytokine with chemokine activity [18]. As a chemokine, it is also involved in the inflammatory response mediated by macrophages. We used the same type of ELISA assay to measure LPS-induced CXCL1 production in the presence or absence of MSO and found no effect (Fig. 4c). LPS was required to induce CXCL1 production; untreated cells or cells treated with MSO alone (minus-LPS) did not produce detectable levels of CXCL1. The effects we see on production of TNFα and IL-6 therefore appear to be specific, and not a result of some nonspecific downregulation of the overall response to LPS.

Finally, to rule out other nonspecific effects of MSO on cell viability such as perturbations in osmotic pressure and plasma membrane integrity, we treated cells with a range of MSO concentrations from 1 to 100 mM and determined cytotoxicity by measuring lactate dehydrogenase activity released into the culture medium [19]. MSO concentrations of up to 100 mM were non-toxic in this assay (Table 1).

**Table 1:** Lactate Dehydrogenase Activity

	Triton X-100	dH_2_O	1 mM MSO	10 mM MSO	20 mM MSO	50 mM MSO	100 mM MSO
OD (490 nm)	0.800	0.04	0.04	0.05	0.03	0.03	0.03
% Cytotoxicity	100	0	0	0	0	0	0

Lactate dehydrogenase (LDH) activity in the medium of cells treated with 1 to 100 mM MSO. Cells were treated with concentrations of MSO ranging from 1 to 100 mM for 2 h or left untreated. Afterwards, cells were treated with Triton X-100 (positive control), or dH_2_O (negative control), and incubated for an additional 45 min before LDH activity in the medium was assessed. LDH activity was completely absent in the medium of cells treated with up to 100 mM MSO, indicating that MSO was not cytotoxic. 100 mM MSO had no inhibitory effect on purified, recombinant LDH (not shown)(See materials in methods for a more complete description of this assay)

### Role of medium glutamine on MSO transport and its effect on IL-6 production

**Fig. 5:**
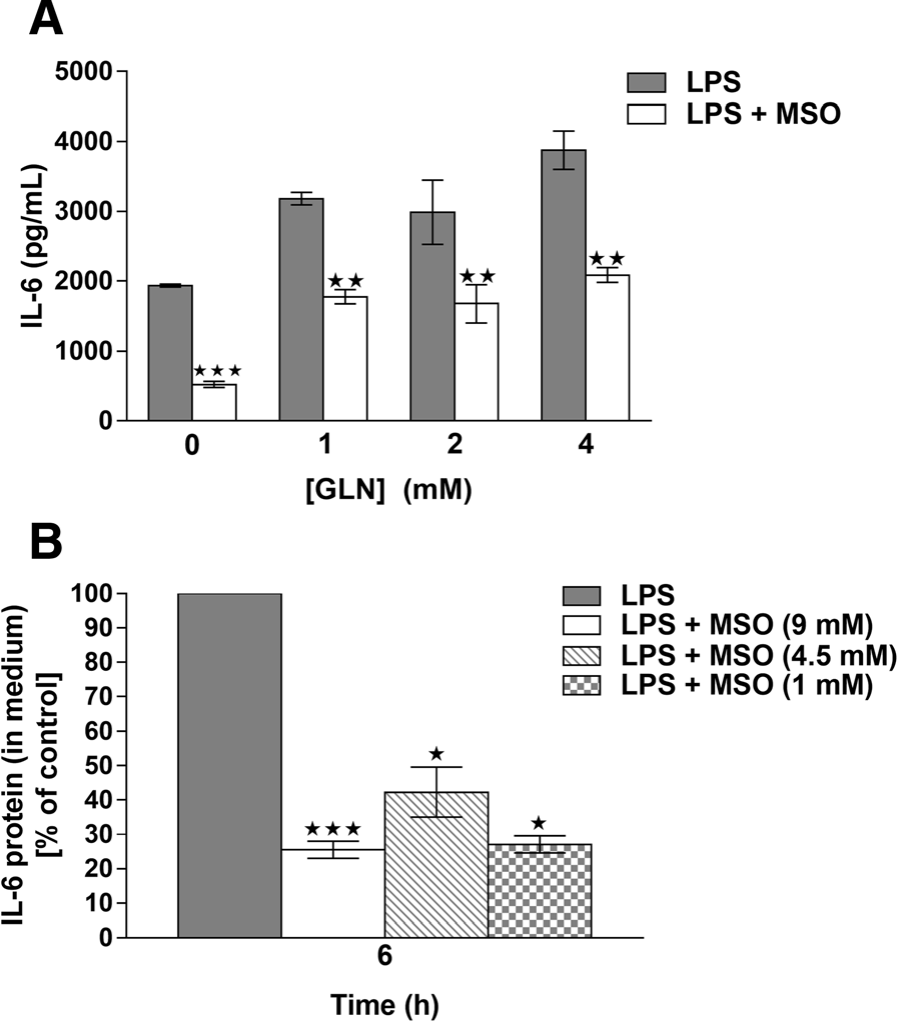
Effect of medium glutamine on IL-6 response to LPS and MSO. (**a**) Peritoneal macrophages were prepared as previously described but were incubated in medium glutamine concentrations ranging from 0 to 4 mM one hour prior to the addition of LPS. As indicated, cells were also treated with 9 mM MSO. At 6 h after LPS treatment, IL-6 in the culture medium was assayed by ELISA. (s ± SEM, *n* = 6) (**b**) Peritoneal macrophages were treated with either 1, 4.5, or 9 mM MSO in medium lacking glutamine for 1 h prior to a 6-h exposure to LPS. Control values were normalized to 100%, and average values from treated samples were plotted in comparison to those controls. All 3 of the tested MSO concentrations yielded significant reductions in medium IL-6, with no significant difference between the % reduction of IL-6 between any of the concentrations

MSO is a glutamine/glutamate/methionine analogue, and it has been demonstrated that glutamine and methionine can compete with MSO for transport into cells in different prokaryotes [20, 21]. Additionally, glutamine is known to enhance the function of immune cells [22]. Levels of medium glutamine and cellular glutamine metabolism are therefore complicating factors for in vitro cell culture experiments involving MSO. The experiments described above were all carried out in medium containing 2 mM glutamine. We therefore attempted to dissect the role of medium glutamine in the observed responses by first measuring LPS-triggered production of IL-6 (+/− MSO) in medium containing different concentrations of glutamine (Fig. 5a). When glutamine was removed from the medium, LPS-stimulated IL-6 secretion by isolated peritoneal macrophages was reduced by approximately 50% compared to production in medium containing glutamine, commensurate with observations from other laboratories. More importantly, the effect of 9 mM MSO was more pronounced in the absence of medium glutamine. The effect of MSO on IL-6 production was the same in media containing 1, 2, and 4 mM glutamine.

We then tested the effect, in the absence of glutamine, of the lower concentrations of MSO (4.5 mM and 1 mM), that previously had little or no effect on IL-6 production in the presence of glutamine (see Fig. 1b). Figure 5b shows that when glutamine was absent, all 3 of the tested MSO concentrations yielded significant reductions in medium IL-6, 6 h after the cells were stimulated with LPS, with no significant difference between the % reduction of IL-6 between any of the concentrations.

This increase in potency was reversed by the addition of 0.5 mM glutamine to the culture medium (data not shown). The fact that MSO potency increases substantially in the absence of glutamine is most likely explained by the presence of a common transport system for MSO and glutamine, as was seen for *S. enterica* [20], although competition between MSO and glutamine for transport has not been studied in murine macrophages.

### Role of glutamine synthetase in MSO mediated cytokine suppression

It is apparent that extracellular glutamine enhances LPS-stimulated IL-6 production in vitro, but it is not clear to what extent, if any, that MSO is acting to inhibit IL-6 secretion through inhibiting the intracellular production of glutamine, because – aside from these experiments measuring dependence on glutamine concentration - in all of our other experiments extracellular glutamine (2 mM) is present in the medium, and MSO concentrations above its K_i_ for glutamine synthetase still inhibit cytokine production. We feel it is therefore unlikely that the effect depends on MSO targeting glutamine synthetase. Nonetheless, we carried out an additional test of the role of GS inhibition in the observed anti-inflammatory effects of this drug by testing different stereoisomers of MSO. Commercial preparations of MSO contain a racemic mixture of two diastereomers – L,*S* and L,*R*, but only the L,*S* isomer inhibits glutamine synthetase activity [23].

To validate the purity of the isomers and to establish GS inhibition, we tested the effect of purified formulations of both the L,*R* and L,*S* diastereomers of MSO, along with the commercial racemic mixture, on mouse liver GS activity using an established GS enzyme assay [24]. We treated mouse liver homogenates with either 1 mM of the racemic mixture, or 0.5 mM of each purified isomer. As was first shown in 1969 [3]we found that the L,*S* isomer inhibited glutamine synthetase, and the L,*R* did not (not shown).

**Fig. 6:**
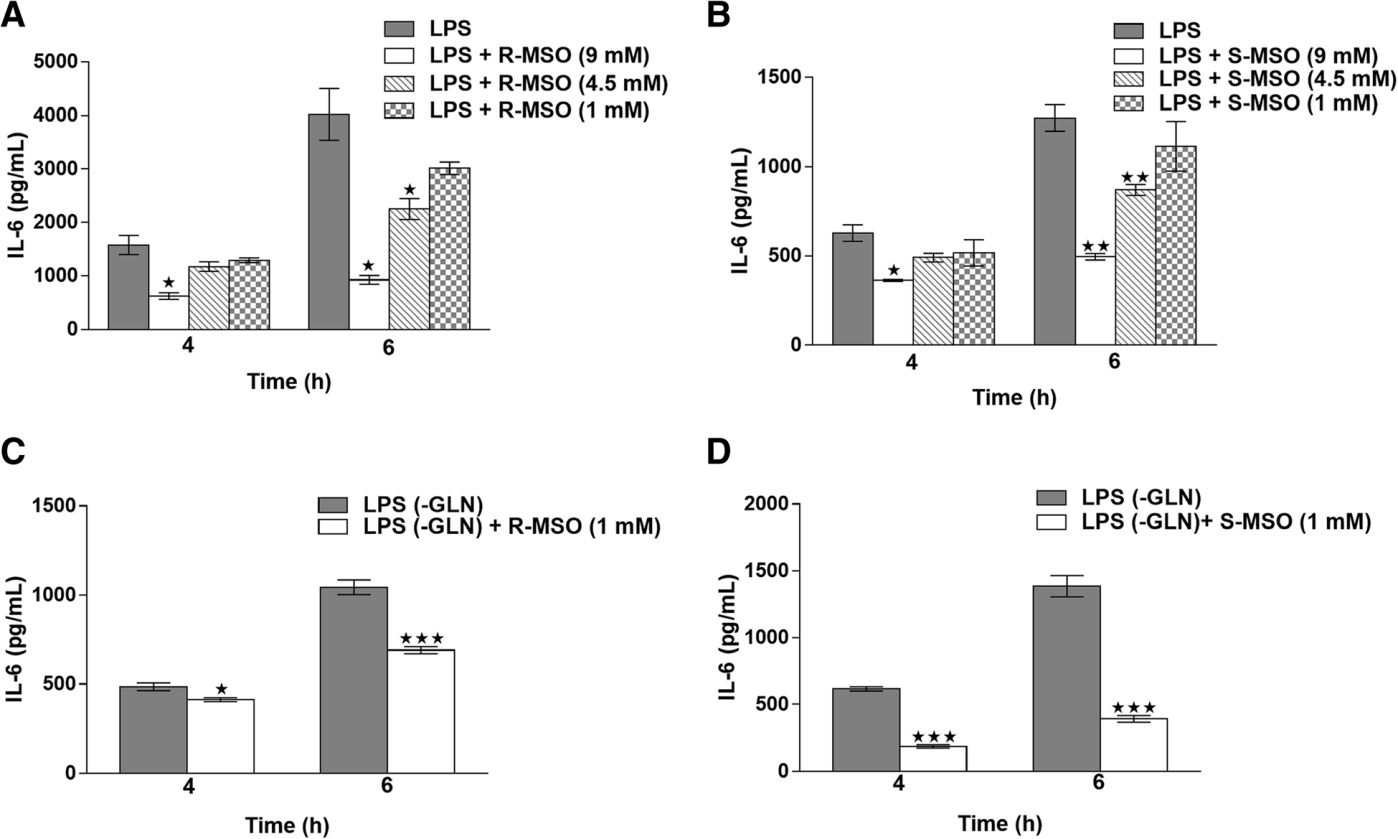
Concentration dependent effect of purified MSO isomers on IL-6 secretion (s ± SEM). Under normal medium conditions (2 mM glutamine), peritoneal macrophages were exposed to either 1, 4.5, or 9 mM of *R*-MSO (**a**) or *S*-MSO (**b**) one hour prior to the addition of LPS. At 4 and 6 h after LPS addition, the medium was harvested and IL-6 was quantified using ELISA. As observed with the racemic mixture, only 9 mM of either isomer caused significant decreases in IL-6 at both time points tested. However, 4.5 mM of either L,*R* or L,*S* MSO caused a significant decrease in IL-6 after 6 h of LPS stimulation. Removing glutamine from the culture medium resulted in an increase of potency of both isomers, as L,*R* (**c**) and L,*S* (**d**) MSO at a concentration of 1 mM significantly reduced IL-6 secretion in the culture medium at both time points tested

We performed experiments using purified formulations of both L,*R* and L,*S* diastereomers of MSO in normal glutamine-containing culture medium (Fig. 6a and b). Both isomers showed a similar dose-dependent response, with 9 mM significantly reducing IL-6 at both time points, whereas 4.5 mM only showed significant IL-6 reduction at 6 h, and 1 mM was ineffective at both time points. As one would expect if glutamine synthetase were the sole target, the L,*S* isomer significantly reduced IL-6 production at both 4 and 6 h after cells were stimulated with LPS (Fig. 6b). However, this effect could only be achieved using 9 mM of purified isomer, twice the concentration present in a racemic mixture, assuming a 50:50 mix of diastereomers. Strikingly, under normal medium conditions, the L,*R* isomer at 9 mM was also shown to independently reduce IL-6 in the culture medium to a significant degree at both time points tested (Fig. 6a). Both isomers therefore inhibit the in vitro cytokine response seen in isolated macrophage preparations. Figure 6a and b illustrate the variability in response from different preparations of macrophages made from different batches of mice. Although the percent inhibition by MSO is consistent across all experiments, the LPS response of the positive controls (minus-MSO) can vary 2–3 fold depending on the batch of mice and the age of the mice. It also varies because the harvesting of macrophages involves peritoneal lavage, which sometimes harvests a significant number of non-macrophage cells. These differences can result in plating different numbers of actual macrophages, even though the number of cells plated is the same.

When glutamine was removed from the culture medium 1 h prior to the addition of LPS, L,*R*-MSO (Fig. 6c) and L,*S*-MSO (Fig. 6d) at a concentration of 1 mM, significantly reduced IL-6 in the culture medium at both 4 and 6 h after LPS exposure, as was the case with the racemic mixture as seen in Fig. 5. Significantly, 4.5 mM of either isomer was ineffective alone in normal culture medium but was effective in reducing IL-6 secretion when combined as a 9 mM racemic mixture. The fact that the L,*R* isomer significantly reduces IL-6 production but does not inhibit GS, combined with the fact that MSO can inhibit cytokine production even in the presence of substantial medium glutamine, strongly suggests that glutamine synthetase is not the most important functional target that inhibits cytokine production in the presence of MSO, if in fact it is a functional target at all.

This in vitro activity of the L,*R* isomer led us to test its activity in vivo, in the same mouse model for liver failure used previously, to show that racemic MSO could protect mice against death from liver failure resulting from an inflammatory immune response(8). We repeated those experiments using the purified isomers, and the results are shown in Table 2. Both isomers were capable of significantly increasing survival in mice injected with LPS and D-Galactosamine to induce fatal liver failure caused by an inflammatory immune response.

**Table 2:** MSO increases survival in a mouse model of inflammatory liver failure

	Alive	Dead	Total (*n*)	Percent Survival (%)	*p* ^*a*^
Saline	3	19	22	14	–
L,*S* MSO (25 mg/kg)	19	9	28	68	0.0002
L,*R* MSO (25 mg/kg)	18	12	30	60	0.0013

24 h survival analysis of mice injected with saline, L,*R* MSO (25 mg/kg), or L,*S* MSO (25 mg/kg) 3 h prior to injection of LPS (20 μg/kg) and D-Galactosamine (800 mg/kg). *a* = determined by Fisher’s exact test

## Discussion

The production of IL-6 and TNFα is essential in mediating the innate immune response to infection [25, 26], as well as regulating other important homeostatic processes such as wound healing [27] and sleep [28, 29]. However, during acute inflammatory crises, overproduction of these and other cytokines can compromise the self-limiting ability of innate immunity and can result in a damaging and occasionally fatal outcome. As such, serum IL-6 levels were shown to be highly valuable in the diagnosis of infection and the prognosis of critically ill ICU patients [30, 31]. Macrophages are the main source of these cytokines, which act in concert to initiate the acute phase response by hepatocytes [32], to increase vascular permeability [33], and to amplify inflammatory responses through a variety of immune cell types. TNFα and IL-6 collectively exacerbate the immune response and its effects on multiple organ systems.

MSO is a well-characterized inhibitor of glutamine synthetase [2, 3, 34]. Our previously published studies on the anti-inflammatory effects of this drug in vivo raised the obvious question of how glutamine synthetase inhibition might be responsible for the observed effects. The in vitro studies described here have enabled us to dissect this process in additional and greater detail. These studies have demonstrated that glutamine plays an important role in both the overall cytokine response –shown previously [35, 36] – and that glutamine levels can modulate how well MSO inhibits that cytokine response.

When we substituted glutamine-free medium 1 h prior to the addition of LPS, we observed a 50% decrease of IL-6 in the culture media compared to cells treated with LPS in medium containing glutamine. The combination of MSO treatment, which decreases glutamine synthetase activity, along with depletion of extracellular glutamine caused the largest decrease in IL-6 secretion that we observed in our experiments (Fig. 5a), suggesting that the anti-inflammatory mechanism might involve glutamine deprivation. Since those results suggest that glutamine in the medium might compete with MSO for transport into the cell, they raise the possibility that, conversely, MSO might compete with glutamine for uptake, albeit weakly, since it takes 9 mM MSO to show an effect in the presence of 2 mM glutamine. The anti-inflammatory effect might therefore involve, to some extent, MSO inhibiting cellular glutamine uptake and thereby reducing the cytokine response.

However, the demonstration that 1) high levels of MSO can inhibit the response even in the presence of 2 mM medium glutamine (conditions where glutamine synthetase would not be needed), and 2) the L,*R* isomer has anti-inflammatory activity on its own, despite not inhibiting glutamine synthetase, means that there is another target for this drug to affect the inflammatory response. MSO may therefore be influencing some aspect of glutamine metabolism important to cytokine production, either by inhibiting glutamine synthetase, inhibiting glutamine transport, or acting on some other unknown target that might or might not involve glutamine metabolism, or some combination of these two potential targets.

As analogues of glutamine, glutamate, and methionine, either or both isomers could be binding to a receptor or transporter for any of these amino acids and exerting their effects through a variety of signaling pathways. These important questions are not addressed by these studies, but answering them could identify a target or targets that could be useful in treating the deleterious side effects of a cytokine storm resulting from an inflammatory immune response.

## Conclusions

1) MSO treatment reduces the LPS-stimulated production of IL-6 and TNFα in vitro, in agreement with our previously published in-vivo results. 2) This effect is specific and not the result of toxicity or overall downregulation of the response to LPS, 3) MSO does not act on transcription, but may be acting on translation or on cytokine turnover and 4) the L,*R* isomer of MSO, which does not inhibit glutamine synthetase, has the same anti-inflammatory activity as the L,*S* isomer, and independently increases survival significantly in an established mouse model of acute liver failure.

If glutamine synthesis is the sole limiting factor in IL-6 production, the L,*R* isomer should not have any activity in these assays. Although it appears possible that glutamine and MSO compete for transport, as the concentration needed to reduce IL-6 by both isomers decreased 10-fold in the absence of extracellular glutamine, such competition would not account for the effects of both isomers of MSO on the cytokine response in medium lacking glutamine. Preliminary experiments in our lab measuring the IL-6 response have demonstrated EC_50_ values of sub-millimolar concentrations of MSO in medium lacking glutamine (not shown). Therefore, it appears that the anti-inflammatory effects of MSO involve some target besides, or perhaps in addition to, glutamine synthetase or glutamine transport. Future attempts to identify this target would therefore also provide a target for therapies to treat diseases involving potentially damaging cytokine responses.

## References

[1] Sellinger OZ, Weiler P,J (1963). The nature of the inhibition in vitro of cerebral glutamine Synthetase by the Convulsant, methionine Sulfoximine. Biochem Pharmacol.

[2] Ronzio RA, Rowe WB, Meister A (1969). Studies on the mechanism of inhibition of glutamine synthetase by methionine sulfoximine. Biochemistry.

[3] Manning JM, Moore S, Rowe WB, Meister A (1969). Identification of L-methionine S-sulfoximine as the diastereoisomer of L-methionine SR-sulfoximine that inhibits glutamine synthetase. Biochemistry.

[4] Brusilow WS, Peters TJ (2017). Therapeutic effects of methionine sulfoximine in multiple diseases include and extend beyond inhibition of glutamine synthetase. Expert Opin Ther Targets.

[5] Brusilow SW, Koehler RC, Traystman RJ, Cooper AJ (2010). Astrocyte glutamine synthetase: importance in hyperammonemic syndromes and potential target for therapy. Neurotherapeutics.

[6] Ghoddoussi F, Galloway MP, Jambekar A, Bame M, Needleman R, Brusilow WS (2010). Methionine sulfoximine, an inhibitor of glutamine synthetase, lowers brain glutamine and glutamate in a mouse model of ALS. J Neurol Sci.

[7] Brusilow WS (2017). Identification of the isomer of methionine sulfoximine that extends the lifespan of the SOD1 G93A mouse. Neurosci Lett.

[8] Jambekar AA, Palma E, Nicolosi L, Rasola A, Petronilli V, Chiara F, Bernardi P, Needleman R, Brusilow WS (2011). A glutamine synthetase inhibitor increases survival and decreases cytokine response in a mouse model of acute liver failure. Liver Int.

[9] Tanaka T, Narazaki M, Kishimoto T (2014). IL-6 in inflammation, immunity, and disease. Cold Spring Harb Perspect Biol.

[10] Esposito E, Cuzzocrea S (2009). TNF-alpha as a therapeutic target in inflammatory diseases, ischemia-reperfusion injury and trauma. Curr Med Chem.

[11] Zhang X, Goncalves R, Mosser DM: The isolation and characterization of murine macrophages. *Curr Protoc Immunol* 2008, Chapter 14:Unit 14 11. 10.1002/0471142735.im1401s83 PMC2834554 19016445

[12] Simpson AE, Tomkins PT, Cooper KL (1997). An investigation of the temporal induction of cytokine mRNAs in LPS-challenged thioglycollate-elicited murine peritoneal macrophages using the reverse transcription polymerase chain reaction. Inflamm Res.

[13] Livak KJ, Schmittgen TD (2001). Analysis of relative gene expression data using real-time quantitative PCR and the 2(−Delta Delta C(T)) method. Methods.

[14] Hines IN, Wheeler MD (2004). Recent advances in alcoholic liver disease III. Role of the innate immune response in alcoholic hepatitis. Am J Physiol Gastrointest Liver Physiol.

[15] DeMarco V, Dyess K, Strauss D, West CM, Neu J (1999). Inhibition of glutamine synthetase decreases proliferation of cultured rat intestinal epithelial cells. J Nutr.

[16] Abusneina A, Gauthier ER (2016). Ammonium ions improve the survival of glutamine-starved hybridoma cells. Cell Biosci.

[17] Dadsetan S, Kukolj E, Bak LK, Sorensen M, Ott P, Vilstrup H, Schousboe A, Keiding S, Waagepetersen HS (2013). Brain alanine formation as an ammonia-scavenging pathway during hyperammonemia: effects of glutamine synthetase inhibition in rats and astrocyte-neuron co-cultures. J Cereb Blood Flow Metab.

[18] Sawant KV, Poluri KM, Dutta AK, Sepuru KM, Troshkina A, Garofalo RP, Rajarathnam K (2016). Chemokine CXCL1 mediated neutrophil recruitment: role of glycosaminoglycan interactions. Sci Rep.

[19] Reid VC, Brabbs CE, Mitchinson MJ (1992). Cellular damage in mouse peritoneal macrophages exposed to cholesteryl linoleate. Atherosclerosis.

[20] Hentchel KL, Escalante-Semerena JC (2015). In Salmonella enterica, the Gcn5-related acetyltransferase MddA (formerly YncA) acetylates methionine sulfoximine and methionine sulfone, blocking their toxic effects. J Bacteriol.

[21] Singh AK, Syiem MB, Singh RS, Adhikari S, Rai AN (2008). A common transport system for methionine, L-methionine-DL-sulfoximine (MSX), and phosphinothricin (PPT) in the diazotrophic cyanobacterium Nostoc muscorum. Curr Microbiol.

[22] Newsholme P: Why is L-glutamine metabolism important to cells of the immune system in health, postinjury, surgery or infection? J Nutr 2001, 131(9 Suppl):2515S–2522S; discussion 2523S–2514S. 10.1093/jn/131.9.2515S 11533304

[23] Rowe WB, Meister A (1970). Identification of L-methionine-S-sulfoximine as the convulsant isomer of methionine sulfoximine. Proc Natl Acad Sci U S A.

[24] Meister A (1985). Glutamine synthetase from mammalian tissues. Methods Enzymol.

[25] Bradley JR (2008). TNF-mediated inflammatory disease. J Pathol.

[26] Scheller J, Chalaris A, Schmidt-Arras D, Rose-John S (2011). The pro- and anti-inflammatory properties of the cytokine interleukin-6. Biochim Biophys Acta.

[27] Barrientos S, Stojadinovic O, Golinko MS, Brem H, Tomic-Canic M (2008). Growth factors and cytokines in wound healing. Wound Repair Regen.

[28] Krueger JM (2008). The role of cytokines in sleep regulation. Curr Pharm Des.

[29] May U, Schiffelholz T, Baier PC, Krueger JM, Rose-John S, Scheller J (2009). IL-6-trans-signalling increases rapid-eye-movement sleep in rats. Eur J Pharmacol.

[30] Takahashi W, Nakada TA, Yazaki M, Oda S (2016). Interleukin-6 levels act as a diagnostic marker for infection and a prognostic marker in patients with organ dysfunction in intensive care units. Shock.

[31] Hou T, Huang D, Zeng R, Ye Z, Zhang Y (2015). Accuracy of serum interleukin (IL)-6 in sepsis diagnosis: a systematic review and meta-analysis. Int J Clin Exp Med.

[32] Castell JV, Gomez-Lechon MJ, David M, Andus T, Geiger T, Trullenque R, Fabra R, Heinrich PC (1989). Interleukin-6 is the major regulator of acute phase protein synthesis in adult human hepatocytes. FEBS Lett.

[33] Hofmann S, Grasberger H, Jung P, Bidlingmaier M, Vlotides J, Janssen OE, Landgraf R (2002). The tumour necrosis factor-alpha induced vascular permeability is associated with a reduction of VE-cadherin expression. Eur J Med Res.

[34] Jeitner TM, Cooper AJ (2014). Inhibition of human glutamine synthetase by L-methionine-S,R-sulfoximine-relevance to the treatment of neurological diseases. Metab Brain Dis.

[35] Murphy C, Newsholme P (1999). Macrophage-mediated lysis of a beta-cell line, tumour necrosis factor-alpha release from bacillus Calmette-Guerin (BCG)-activated murine macrophages and interleukin-8 release from human monocytes are dependent on extracellular glutamine concentration and glutamine metabolism. Clin Sci (Lond).

[36] Yassad A, Lavoinne A, Bion A, Daveau M, Husson A (1997). Glutamine accelerates interleukin-6 production by rat peritoneal macrophages in culture. FEBS Lett.

